# Bacterial expression, correct membrane targeting and functional folding of the HIV-1 membrane protein Vpu using a periplasmic signal peptide

**DOI:** 10.1371/journal.pone.0172529

**Published:** 2017-02-22

**Authors:** Arpan Deb, William A. Johnson, Alexander P. Kline, Boston J. Scott, Lydia R. Meador, Dustin Srinivas, Jose M. Martin-Garcia, Katerina Dörner, Chad R. Borges, Rajeev Misra, Brenda G. Hogue, Petra Fromme, Tsafrir S. Mor

**Affiliations:** 1 School of Life Sciences, Arizona State University, Tempe, Arizona, United States of America; 2 The Biodesign Center for Immunotherapy, Vaccines, and Virotherapy, The Biodesign Institute, Arizona State University, Tempe, Arizona, United States of America; 3 School of Molecular Sciences, Arizona State University, Tempe, Arizona, United States of America; 4 The Biodesign Center for Applied Structural Discovery, The Biodesign Institute, Arizona State University, Tempe, Arizona, United States of America; 5 The Biodesign Center for Personal Diagnostics, The Biodesign Institute, Arizona State University, Tempe, Arizona, United States of America; University Hospital Tuebingen, GERMANY

## Abstract

Viral protein U (Vpu) is a type-III integral membrane protein encoded by Human Immunodeficiency Virus-1 (HIV- 1). It is expressed in infected host cells and plays several roles in viral progeny escape from infected cells, including down-regulation of CD4 receptors. But key structure/function questions remain regarding the mechanisms by which the Vpu protein contributes to HIV-1 pathogenesis. Here we describe expression of Vpu in bacteria, its purification and characterization. We report the successful expression of PelB-Vpu in *Escherichia coli* using the leader peptide pectate lyase B (PelB) from *Erwinia carotovora*. The protein was detergent extractable and could be isolated in a very pure form. We demonstrate that the PelB signal peptide successfully targets Vpu to the cell membranes and inserts it as a type I membrane protein. PelB-Vpu was biophysically characterized by circular dichroism and dynamic light scattering experiments and was shown to be an excellent candidate for elucidating structural models.

## Introduction

Structural analysis of membrane proteins has traditionally been bottlenecked by the lack of sufficiently large amounts of pure, properly folded and functional membrane proteins. The issue is particularly important considering the fact that membrane proteins constitute a significant proportion of clinical drug targets.

The viral protein U (Vpu) was identified as a product of the *vpu* open reading frame from the human immunodeficiency virus-1 (HIV-1) which runs as a 16 kDa protein on a SDS-polyacrylamide gel [1]. It is a type III transmembrane protein 77–86 amino acids in length, depending on the group and subtype of HIV-1. Vpu is not expressed by HIV-2, but is present in some simian immunodeficiency viruses (SIVs) [2]. It has a very short N-terminal domain, a transmembrane domain and a longer cytoplasmic domain, which has two predicted α-helical domains, separated by a hinge region. The hinge region contains two potential casein kinase II sites [3]. NMR characterization of the cytoplasmic domain of Vpu indicates that it exhibits a great deal of structural flexibility [4].

Down-regulation of CD4 receptors in infected host cells is of prime importance for the virus and it accomplishes this through the Nef and Vpu proteins. Vpu not only retains CD4 in the endoplasmic reticulum, but also induces CD4 degradation by employing a variant pathway of the ERAD machinery [5]. Vpu retains CD4 in the ER by virtue of interactions of the transmembrane domains. The conserved tryptophan residue (Trp22 in NL4-3 strain Vpu sequence) in the transmembrane domain of Vpu plays a role in inhibiting the dislocation of the CD4 from the ER membrane to the cytosol for degradation. Val20 and Ser23 have also been identified to play a role in CD4 retention in the ER [6]. The cytosolic domain of Vpu recruits ubiquitination factors, which ubiquitinate the cytoplasmic domain of CD4 at multiple lysine and serine/threonine residues to mark CD4 for proteasomal degradation and also further contribute in retention of CD4 in the ER [5].

The transmembrane domain of Vpu is involved in its association with tetherin, leading to the ubiquitin-dependent degradation of the latter, which in turn reduces the incorporation of tetherin into nascent virions. According to the suggested mechanism, phosphorylated Vpu recruits a SCG TRCP1/2 E3 ligase complex that ubiquitinates the cytoplasmic tail of tetherin at multiple residues. A motif in the second α-helix in the cytoplasmic domain of Vpu has been identified as responsible for efficient counteraction of tetherin [7].

Vpu has also been implicated in retention of NK, T-cell, B-cell antigen (NTB-A) in the Golgi apparatus by altering the glycosylation pattern in nascent NTM-A. This is a part of a strategy to prevent HIV-1-infected cells from being lysed by NK cells. This function has been traced to the second α-helical region of Vpu in its cytoplasmic domain [8]. In addition, based on similarities with other viral transmembrane proteins, Vpu has also been studied for possessing an ion-channel activity, which could potentially make the host membrane protein more permeable and aid in budding and release of nascent virions. Vpu has been shown to mediate potassium transport when expressed recombinantly in *Saccharomyces cerevisiae* [9].

The 22-residue-long PelB signal sequence was first described in the identification and characterization of *Erwinia carotovora* pectate lyase B gene and its protein product [10]. The expression of the mature form of the protein in *E*. *coli* indicated that the protein translocation machinery and signal peptidase(s) can correctly process and export the protein to the periplasmic space. Since then a wide variety of recombinant soluble proteins have been efficiently targeted to the *E*. *coli* periplasm and also to the extracellular media using this signal peptide like the ligand-binding domains of glutamate receptor’s B and D subunits [11], variable regions of light and heavy chains of antibodies [12, 13], phospholipase D [14], and the C-terminal domain of OmpC [15]. The PelB signal peptide was also used to express a pentameric ion channel protein ELIC with an N-terminal fusion of maltose binding protein, which led to the successful crystallization and x-ray structure determination of ELIC [16]. It was also used to target a fusion of the B subunit of cholera toxin and the membrane proximal region of the gp41 protein of HIV-1, which interacts with the periplasmic side of the inner membrane in *E*. *coli* [17–19].

In this study we report the successful expression of HIV-1 Vpu in *E*. *coli* as a type I membrane protein with PelB signal sequence appended to the amino end of the protein. We also describe the purification and characterization of the target protein towards its suitability for structure determination by X-ray crystallography.

## Results and discussion

### Gene optimization, cloning and expression of PelB-Vpu

Vpu is an important accessory protein involved in HIV-1 pathogenesis. Furthermore, the presence of a transmembrane domain with an intriguing structure-function relationship makes it an attractive target for structural studies. Previous attempts from our laboratory to express this protein were either unsuccessful or resulted in proteins that lacked the necessary purity and stability for crystallization. Attempts were made to express the protein using the P8CBD expression vector described by Luo et al. [20] and also as an N-terminal fusion with the *Bacillus subtilis* protein Mistic (see Kefala and Kwiatkowsk [21] and unpublished data).

**Fig 1:**
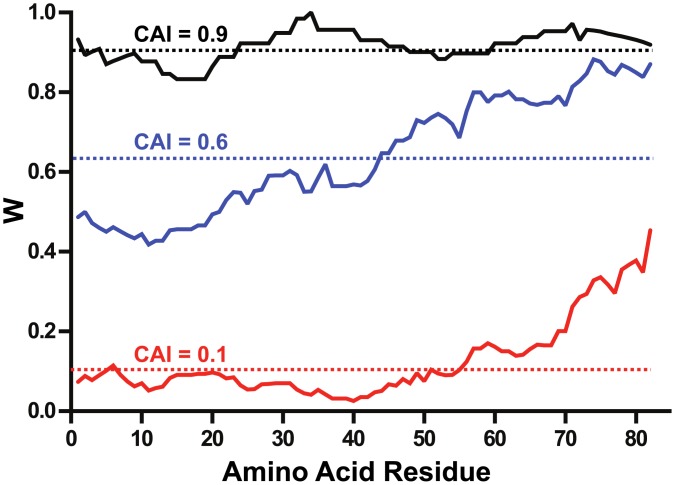
Optimization of the *vpu* gene for expression in *E*. *coli*. Codon usage is presented by plotting the relative adaptiveness, *w*, values (a moving geometric mean with a 10-residue window) as a function of the residue number. Codon usage analysis of the native HIV-1 Vpu gene against the reference set of *E*. *coli* and *H*. *sapiens* are shown as red and blue traces. The codon usage of the *vpu* gene optimized for expression in *E*. *coli* is shown in black. The codon adaptation index (CAI, relative adaptiveness averaged over the entire length of a sequence) for each gene is shown as a broken line.

In this study, unfavorable codons in the native Vpu gene from subtype B HIV-1 were removed and replaced by codons widely used by *E*. *coli* (Fig 1). Codon optimization to increase yield and stability of recombinant proteins has been successfully used in our laboratory for the expression of human butyrylcholinesterase [22] and HIV-1 Gag/dgp41 virus like particles [23] in *Nicotiana benthamiana*. Codon optimization of the Vpu gene has been shown to improve its mRNA stability and magnitude of expression in HeLa cells when transcribed under the control of a CMV promoter [24].

**Fig 2:**
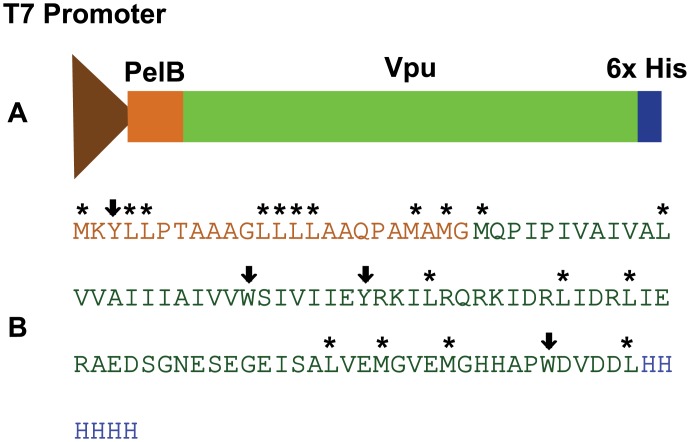
Organization of the expression cassette within the plasmid pTM 875. Expression was driven by a T7 promoter as shown in (**A**). The Vpu gene is flanked by the PelB signal peptide at the N-terminus that directs the protein to the periplasmic space of the bacteria and facilitates integration into the inner membranes, and a 6x- histidine tag at the C- terminus to aid in purification. (**B**) shows the sequence of the PelB signal peptide (in orange), Vpu (in green) and histidine tag (in blue). Chymotrypsin cleavage sites are denoted by down-pointing arrows (⬇) for strong recognition sites and by an asterisk (*) for secondary sites.

The native *vpu* gene is composed of significantly unfavorable codons with respect to the *E*. *coli* codon usage patterns, with a codon adaptation index (CAI) of 0.101. Removal of these unfavorable codons and replacing them with codons more suited to the *E*. *coli* codon usage patterns resulted in an increase of the CAI to 0.916. In the human genome, there is a weak positive correlation between frequency of optimal codons and levels of gene expression [25, 26]. It is interesting to observe that the frequency of non-optimal codons is significantly higher for the N-terminal part of the native protein. There is evidence to suggest that usage of low frequency codons within a coding sequence could be indicative of a genetic instruction that regulates the rate of protein synthesis. This phenomenon could facilitate the formation of some secondary and/or tertiary structure in the nascent polypeptide [27, 28]. But, the idea of the action of codon usage bias to improve translational efficiency remains unconfirmed in mammals [29]. On the other hand, in unicellular organisms like *E*. *coli*, there is a strong correlation between the most frequently used codons and the abundance of tRNAs [30, 31]. Thus, the codon usage pattern can greatly influence the rate and efficiency of translation in *E*. *coli* [32–34], leading to enhanced expression levels of the recombinant membrane protein. Thus, the *E*. *coli* optimized gene was cloned into a pET26(b) expression vector to obtain pTM 875 (Fig 2) with the addition of a C-terminal hexa-histidine tag.

### Expression in *E*. *coli*

**Fig 3:**
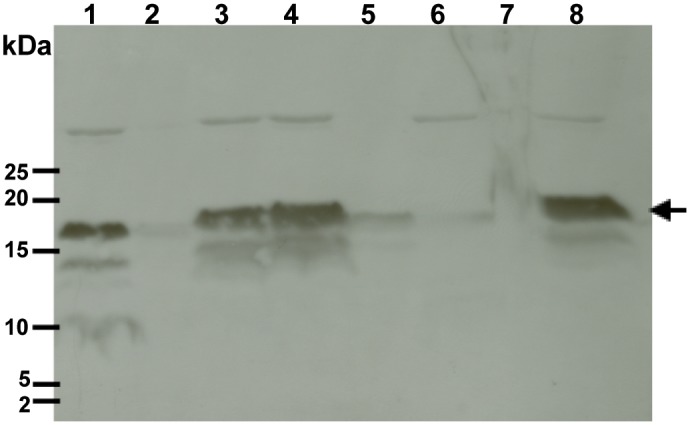
Vpu fractionates with the water insoluble fraction and can be subsequently solubilized by detergents. Whole cell lysates of *E*. *coli* BL21s expressing Vpu were fractionated into non- aqueous (lane 1) and aqueous (lane 2) fractions by centrifugation. The non-aqueous fraction (pellets) were resuspended in extraction buffer containing various detergents (1% final concentration): βDM (lane 3), βDDM (lane 4), CHAPS (lane 5), OG (lane 6), LDAO (lane 7) and DPC (lane 8). Following overnight incubation at 4°C, detergent-soluble proteins were separated from insoluble proteins by centrifugation and fractionated by SDS-PAGE followed by immunoblot analysis using a Vpu-specific antibody. Vpu is indicated by a black arrow.

Bacterial cells harboring pTM 875 were grown to mid-logarithmic phase when 0.1 mM IPTG was added to the culture medium to induce expression of the recombinant PelB-Vpu. At 6 h post induction, cells were harvested and lyzed by a microfluidizer. Following cell lysis, cellular components were fractionated into aqueous and non-aqueous fractions and their proteins resolved by SDS-PAGE. Immunoblot analysis revealed the almost-exclusive presence of PelB-Vpu in the water-insoluble fraction. However, PelB-Vpu could be extracted from the water-insoluble fraction with detergents. Particularly effective in extraction of PelB-Vpu were the maltoside detergents βDM and βDDM and the zwitterionic detergent, DPC (Fig 3). These three detergents have relatively long and flexible hydrophobic chains, which may explain in part the ability of these detergents to effectively extract the highly hydrophobic PelB-Vpu protein. Consistent with this possible explanation is our observation that the shorter-chained OG and CHAPS, whose hydrophobic tail consists of a rigid steroidal ring system, have relatively ineffective extraction power. Long flexible hydrophobic tails may be necessary for successful extraction of PelB-Vpu, but apparently are not sufficient as LDAO performed poorly; LDAO solubilization power may be hampered by a very small hydrophilic head (18 Å^2^ as compared to 59 Å^2^ and 179 Å^2^ of DPC and the two maltoside detergents, respectively) [35–37] and a high octanol/water partition coefficient (log*P* > 5.3) [37]. Future studies involving the systematic testing of detergents with various head groups and alkyl-chain that vary by length may provide a more conclusive explanation and may allow rational selection of the appropriate detergents instead of the current largely empiric exercise. Based on its performance and its suitability for downstream applications, we elected to use βDDM in all subsequent experiments.

### Vpu is targeted to the membranes and inserted as a type I membrane protein

In mammalian cells, the Vpu transmembrane domain (TMD) serves as a signal-anchor to target the protein for insertion into the membrane, but when expressed in bacteria, the TMD does not support this insertion function, resulting in the accumulation and aggregation of the protein in inclusion bodies [38]. The PelB leader sequence targets its cargo protein pectate lyase B of *Erwinia carotovora* into the periplasm via the Sec-dependent pathway [39–41], which is also used for targeting integral-membrane proteins to the inner membrane [42]. While PelB is commonly used to direct recombinant proteins to the periplasm of Gram-negative bacteria, there are not many examples of its application in targeting membrane proteins to the bacterial inner membrane, as was intended here. Because the non-aqueous fraction obtained after cell lysis contained both membranes with their respective membrane proteins and aggregated proteins in the form of inclusion bodies, the question arose as to the partition of the recombinant PelB-Vpu between these two compartments.

**Fig 4:**
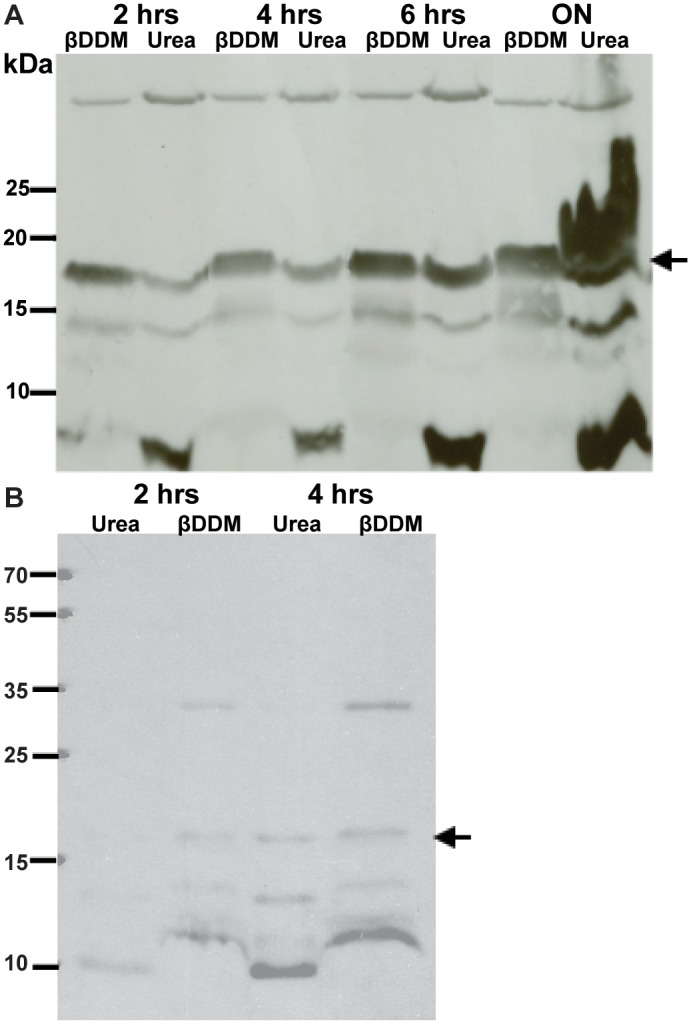
Vpu fractionates with the membrane fraction and inclusion bodies dependent on time post-induction with IPTG. The βDDM extractable fraction increases with time until it reaches saturation around 6 hr (**A**). When the non-aqueous fraction is first treated with urea and then βDDM (**B**), the protein first fractionates in the detergent fraction, implying that protein does integrate in the membranes that is extractable by βDDM. Vpu is indicated by a black arrow.

To answer this question, we have used differential extraction of PelB-Vpu from samples of water-insoluble protein fractions obtained at various times after the beginning of recombinant protein induction. To this end, we used our mild detergent of choice, βDDM, to extract membrane proteins and subjected the residual detergent-insoluble proteins to extraction with 8 M Urea, a chaotropic agent that can solubilize protein inclusion bodies [43–45]. Extractions were carried out at the specified time points following the induction with IPTG. The amount of PelB-Vpu extractable with βDDM increased with time during the first 6 hr post-induction (Fig 4A). However, there was no increase of the amount of PelB-Vpu that could be extracted from homogenization pellets of cells harvested after overnight growth in the presence of IPTG as compared to 6 hr. In striking contrast, the overnight sample had a significant increase in amount of urea-extractable PelB-Vpu. This could be explained by saturation of the bacterial inner membrane by 6 hr post induction and subsequent accumulation of the over-expressed protein in intracellular inclusion bodies.

Urea cannot extract integral membrane proteins embedded in membranes, but such proteins can be extracted by detergents. Conversely, detergent-extractable proteins may be present in inclusion bodies, but such proteins should also be solubilized by urea. To rule out this second possibility, and to further demonstrate that βDDM predominantly extracts PelB-Vpu from the cell membranes rather than from inclusion bodies, we reversed the order of extraction such that the non-aqueous fraction was first subjected to urea extraction followed by βDDM extraction. Most of the protein from cells harvested 2 h and a comparable amount at 4 h after induction was in the βDDM extractable fraction (Fig 4B). This is consistent with results in panel A which indicates that most of the protein localized in the membrane fraction until around 6 h after induction with IPTG, after which the membranes may saturate and the subsequently produced recombinant protein forms aggregates that can be solubilized by urea. Based on this finding, the cells were harvested after 6 hr of IPTG induction for all subsequent experiments.

The PelB sequence allows the insertion of recombinant PelB-Vpu into the membrane, however the presence of two in tandem hydrophobic domains may complicates the membrane topology of the protein. To address this, we next sought to determine *in vivo* whether the protein is oriented with its N-terminus in the periplasm (functionally equivalent to the ER lumen in human cells) and its C-terminus inside the cell (in the cytoplasm).

**Fig 5:**
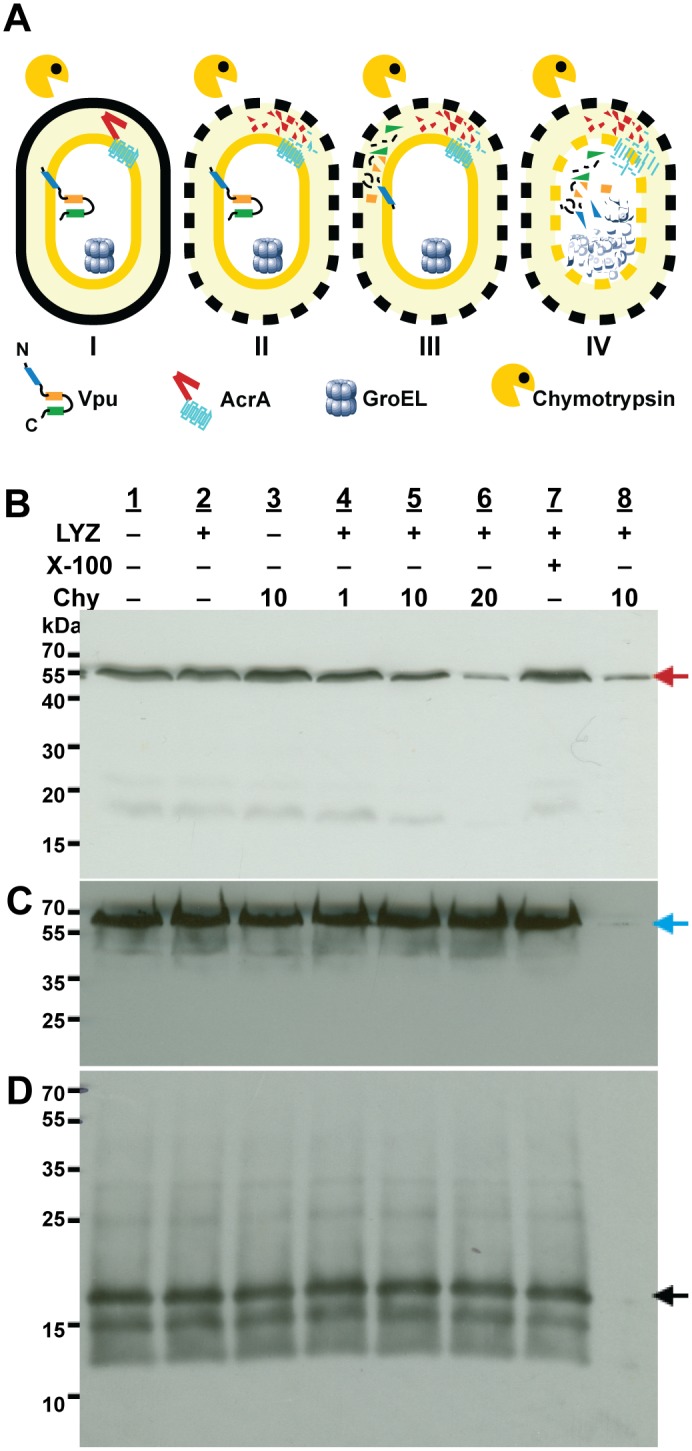
Vpu is inserted into the bacterial inner membrane with its C-terminal domain within the cytoplasm. Panel A depicts the possible outcomes when *E*. *coli* cells (I) and protoplasts (II III and IV) that express Vpu are digested with chymotrypsin alone (I-III) and in the the presence of Triton X-100 (IV). If the C-terminal domain of Vpu is protected by the inner membrane (I and II), it is expected to be resistant to chymotrypsin digestion, similar to the case of the cytoplasmic resident protein GroEL. If the C-terminal domain of Vpu is exposed at the periplasmic space of the cells (III), it is expected to be digested in the same way as AcrA. All three proteins should undergo chymotrypsin cleavage when the inner membranes are permeabilized by the addition of Triton X-100 (IV). *E*. *coli* cells (lanes 1 and 3) and protoplasts (lanes 2 and 4–8) were incubated without chymotrypsin (lanes 1, 2, and 7) or with the indicated concentrations of chymotrypsin (μg/mL); in the presence of 1% Triton X-100 (lanes 7 and 8) or its absence (lanes 1–6). Following treatments, cells were solubilized in the presence of SDS sample buffer and protein samples were resolved by SDS-PAGE followed by immunoblotting with antibodies against the periplasmic domain of the inner-membrane protein AcrA (Panel B, red arrow), cytoplasmic protein GroEL (Panel C, blue arrow) or Vpu C-terminal domain (Panel D, black arrow).

To this end, we treated *E*. *coli* cells expressing PelB-Vpu with lysozyme and subjected the recovered protoplasts to mild chymotrypsin proteolysis. The C-terminal domain of Vpu contains several chymotrypsin proteolytic sites, which should be protected from the protease if the protein is inserted with its C-terminus in the cytoplasm. However, if the orientation of the protein is such that the C-terminus is externally-exposed, it will be cleaved and should not react with the polyclonal antibodies against Vpu. Indeed, the chymotrypsin digestion assay revealed that the C-terminal domain of Vpu is shielded from proteolytic digestion by the *E*. *coli* inner membrane (Fig 5). In contrast, the periplasmic domain of AcrA, a native *E*. *coli* inner-membrane efflux pump, was vulnerable to proteolytic cleavage. (Fig 5B). The cytoplasmic chaperone GroEL, like the cytoplasmic domain of Vpu, was immune to the effects of chymotrypsin (Fig 5C and 5D respectively). Vpu and GroEL could be digested by chymotrypsin only when the integrity of the *E*. *coli* inner membrane was compromised by treatment with the detergent Triton X-100. Our results support the hypothesis that PelB-Vpu is inserted into the membrane with its C-terminal domain located in the cytoplasm.

### Purification of PelB-Vpu

**Fig 6:**
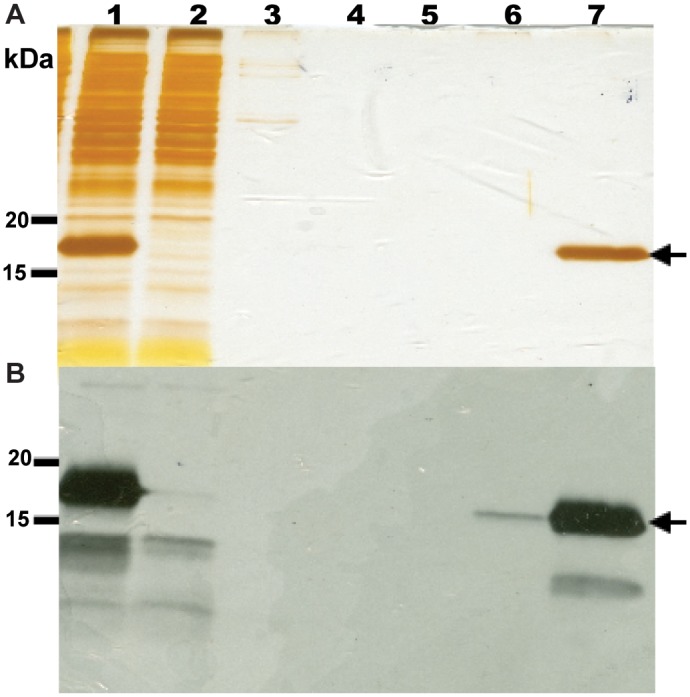
Protein analysis of the talon metal affinity chromatography purification of PelB-Vpu. Protein fractions were subjected to SDS-PAGE followed by silver staining (**A**) and immunoblotting (**B**). Detergent (βDDM)-soluble fraction containing PelB-Vpu (lane 1) was loaded onto a column containing Talon resin that was pre-equilibrated with 20 mM HEPES, pH 7.5, supplemented with 500 mM NaCl, 5 mM imidazole, and 0.02% βDDM. Unbound proteins in the flow through were collected (lane 2) and the column was washed consecutively with four different buffer solutions (20 mM HEPES, pH 7.5): W1 (lane 3, buffer plus 500 mM NaCl), W2 (lane 4, buffer plus 500 mM NaCl and 0.02% βDDM), W3 (lane 5, buffer plus 250 mM NaCl and 0.02% βDDM), and W4 (lane 6, buffer plus 250 mM NaCl, 0.02% βDDM and 10 mM imidazole). Pure PelB-Vpu was eluted with W4 buffer supplemented with 300 mM imidazole (lane 7, arrows).

**Fig 7:**
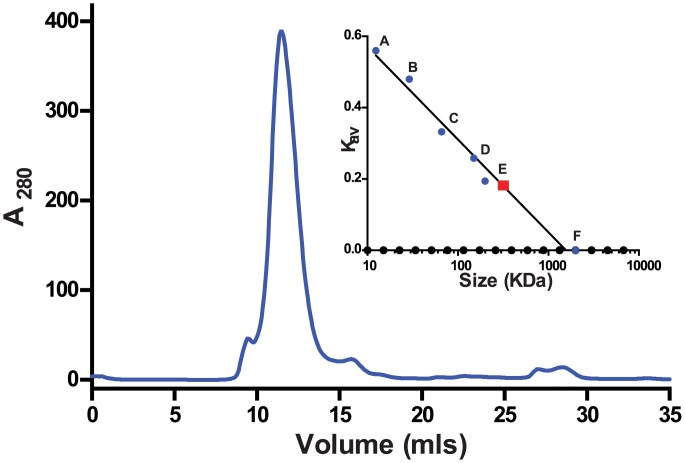
Size-exclusion chromatography of concentrated metal-affinity chromatography-purified PelB-VPU. Eluate from Fig 6 was subjected to SEC-FPLC. Sec resin: Superdex 200 10/300 GL; Flow-rate: 0.5 mL/min, Running buffer: 20 mM HEPES, pH 7.5, 250 mM NaCl, 5% glycerol and 0.02% βDDM. The minor peak at 8 mL corresponded to the void volume, and the major peak at 11.48 mL contained PelB-Vpu. The column was calibrated with cytochrome c (A), carbonic anhydrase (B), albumin (C), alcohol dehydrogenase (D), β-amylase (E) and blue dextran (F) and a standard curve was obtained as shown in the inset. The size of the Pel-B-Vpu- βDDM complex was estimated to be 315 kDa.

Crude detergent extracts contain several protein bands that are recognized by the anti-VPU antibody we used (Figs 3 and 4). Very small fragments are likely representing degradation products, but the nature of the higher molecular mass bands is not clear. To purify PelB-Vpu from cellular proteins and small protein fragments, we employed metal affinity chromatorgraphy. Immobilized metal affinity purification of membrane protein-detergent complexes can be challenging in terms of amount and purity of the enriched protein. The choice of detergent and buffers can have some effect on the efficiency of the purification procedure [46, 47]. The conformation of the protein could also play a role in retention of the protein by the affinity resin [48, 49]. The folding state can also dictate the placement of the affinity tag in the protein and its accessibility to the metal resin [50]. Towards our effort to isolate His-tagged PelB-Vpu from the rest of the cellular biomolecules, the βDDM solubilized fraction was subjected to TALON metal affinity. We observe that the protein in the elution fraction was obtained in a highly pure form (Fig 6A). We were also able to obtain PelB-Vpu protein with minimal loss during the purification procedure in the flow-through and wash steps (Fig 6B). Although the purity of the eluate was demonstrated by silver-stained SDS-PAGE gels, it is also important to analyze the sample for high molecular weight aggregates, which may be undetectable due to the denaturing and reducing nature of SDS-PAGE. With this objective, concentrated IMAC purified protein was analyzed by size exclusion (SEC) chromatography. We observe that the protein eluted as mostly a solitary peak. Void volume aggregates eluted as a distinctly small distinct peak before the major peak, which was easily separated and discarded (Fig 7).

### Size of PelB-Vpu

Our results so far suggest that the fusion of the PelB signal sequence converted Vpu, a Type III membrane protein, into a protein more akin to a Type I membrane protein. However, unlike most Type I proteins, from which the signal peptide is typically cleaved concomitantly to their insertion into the membrane, the apparent molecular mass of the recombinant protein of approximately 17.5 kDa, as estimated from denaturing gels, suggests that the PelB sequence is not cleaved off PelB-Vpu. The predicted molecular mass of Vpu based on its primary sequence including the engineered poly-histidine tag and PelB is 12463.7 Da. If the signal peptide would be cleaved the predicted molecular mass would be is 10,064.7 Da. However, Vpu is known to migrate with apparent MW of ~16 kDa when analyzed by SDS-PAGE [1].

Interestingly, a band appears below the full-length PelB-Vpu (~15 kDa). Following metal-affinity chromatography, we could detect the presence of the lower MW band by immunoblotting but not by silver-staining. The fact the band co-purifies with the full-length PelB-Vpu suggests that the former retains its C-terminal His tag. It is therefore possible that the protein represents a small population of Vpu protein obtained upon cleavage of the PelB leader sequence.

**Fig 8:**
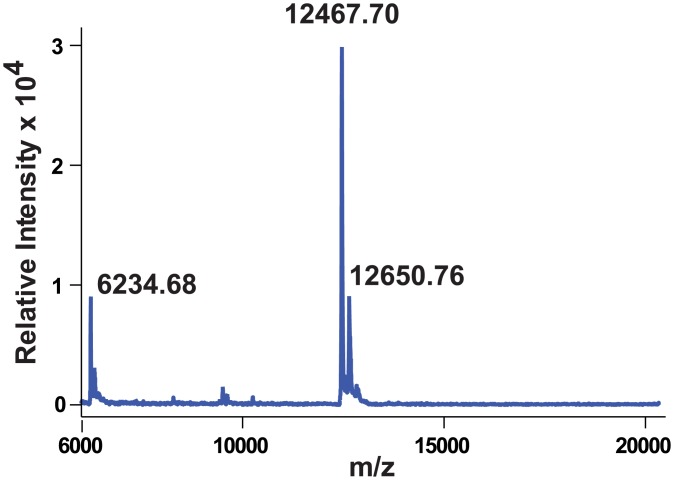
MALDI-TOF spectrum of PelB-Vpu reveals that the purified protein retains the PelB peptide. The major peak corresponding to the MH^+^ form of the protein was detected at 12467.7 Da.

To more accurately determine the size of bacterially-produced PelB-Vpu, we subjected the SEC-FPLC-purified protein to mass spectrometry (MALDI-TOF, Fig 8). The peak representing the MH^+^ form of the protein at 12467.7 Da, corresponds well to the scenario in which the PelB signal peptide remains uncleaved. Inefficient cleavage by periplasmic peptidases could be a result of steric hindrance due to the proximity of the cleavage site and the five residue-long N-terminal ectodomain to the hydrophilic heads of phospholipids in the outer leaflet the plasma membranes of *E*. *coli*. Note that the mass spectrogram does not indicate the presence of the lower molecular mass Vpu, which was likely removed at the SEC-FPLC stage.

The accurate determination of the oligomeric state of a membrane protein in a protein-detergent micelle complex is very difficult. The size of the non-denatured PelB-Vpu within the context of its associated βDDM micelle is shown by size exclusion chromatography to be around 315 kDa (Fig 7). This indicates that PelB-Vpu exists as an oligomer in complex with the βDDM micelles. The size of empty βDDM micelles size is estimated to be 60–70 kDa [51–54], however, it is difficult to predict the size of the βDDM-protein micelles as the detergent micelle “belt” surrounding a membrane protein is larger than an empty micelle.

### Preparations of purified PelB-Vpu are uniform and the protein is folded and stable

**Fig 9:**
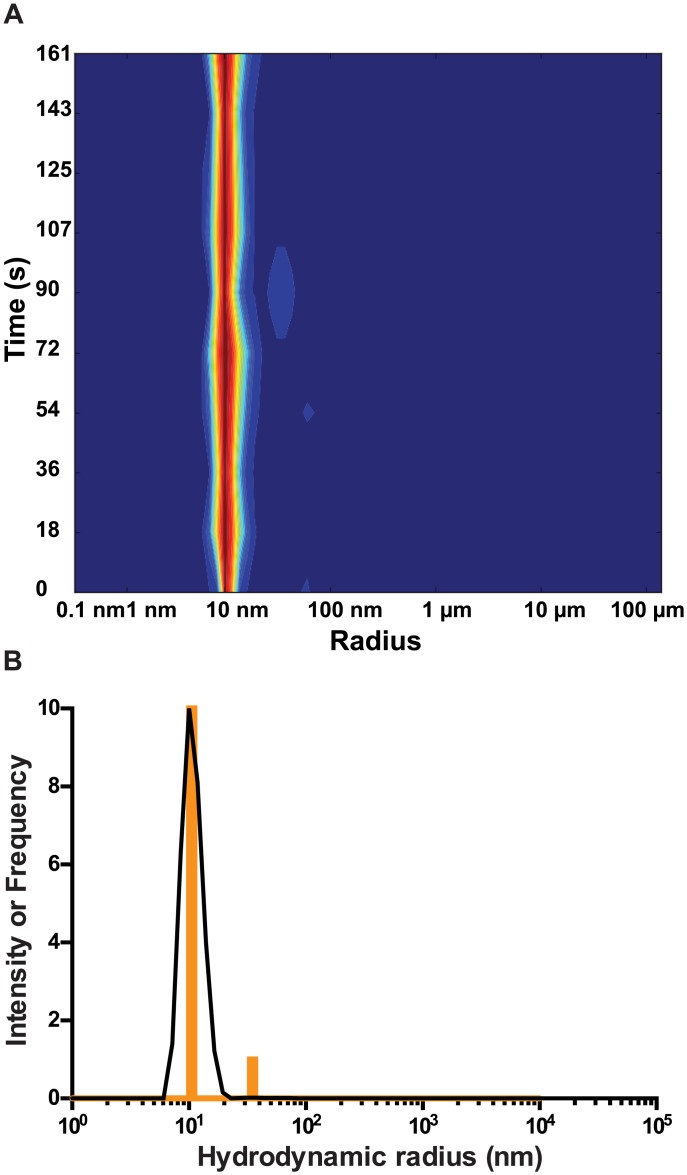
Dynamic light scattering measurements show that PelB-Vpu is monodispersed. *The complex of detergent and PelB-Vpu was detected as a narrow peak at 10 nm*. *A molecular weight of* 332.2 *kDa was predicted for the protein-detergent complex*. Monodispersity of PelB-Vpu (10 mg/mL) is shown by the heat map diagram (A) and a plot of frequency of each particle size estimated from multiple measurements (B).

Dynamic light scattering of purified PelB-Vpu preparations reveals very low dispersity at a concentration of 5 mg/mL and 10 mg/mL with a 10.4±0.1 nm hydrodynamic radius (data not shown and Fig 9). This estimation was fairly consistent upon re-runs of the experiment as is evident by the histogram depicting the frequency of each size estimation over 10 runs (Fig 9B). The hydrodynamic radius corresponds to a protein-detergent complex of about 332.2 kDa, corresponding well to the molecular mass estimates reported above for size-exclusion chromatography.

Circular dichroism (CD) was used to estimate the secondary structure of PelB-Vpu. The CD spectrum of PelB-Vpu displayed two negative peaks at 208 and 222 nm, characteristic of a α-helical protein. Spectra for wavelengths below 200 nm could not be obtained because of interference from the chloride ions in the buffer. Analysis of the PelB-Vpu CD data (in 20 mM HEPES pH 7.5, 250 mM NaCl, 0.02% βDDM, and 5% glycerol) by CONTINLL in the CDPro software package indicated 55.6% right handed helix, 23% left handed helix, 3.3% sheets, 2% turns and 16% random elements, corresponding well to *in silico* analysis with the secondary structure prediction tool, GOR [55], which estimated 65.2% helices in the sequence.

**Fig 10:**
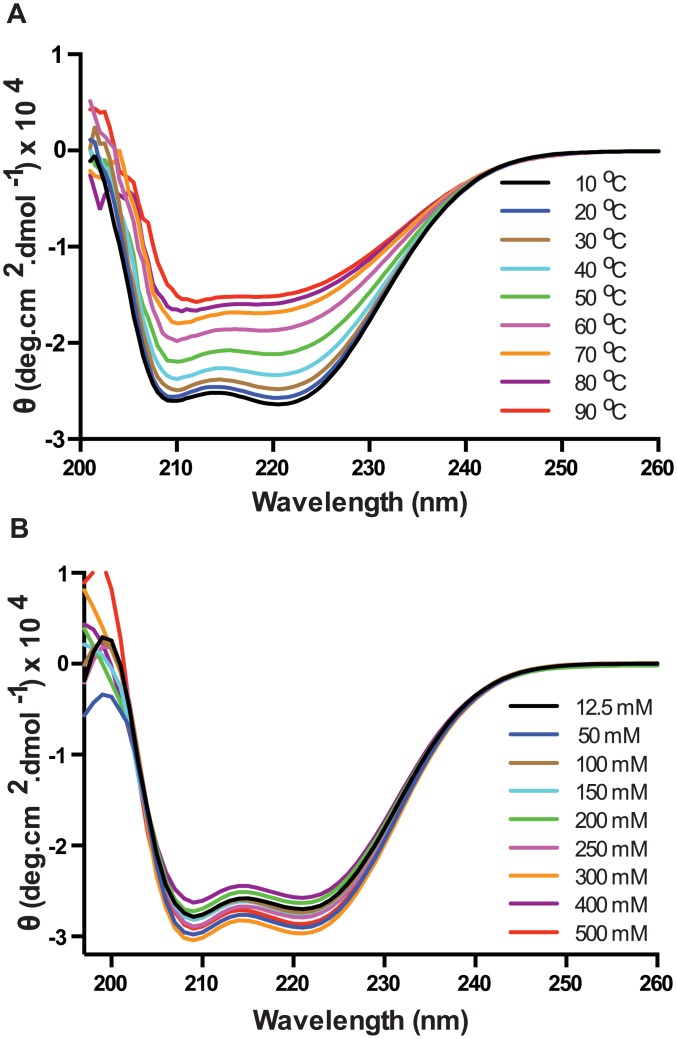
Circular dichroism spectrometry analysis shows that PelB-Vpu is stable (A) at various temperatures up to 40°C and (B) across a wide spectrum of salt concentrations.

Purified PelB-Vpu was observed to be stable up to around 40°C after which a temperature-dependent disintegration of the α-helical secondary structure was observed (Fig 10A). It was also observed that the protein was stable across a wide range of salt concentrations (Fig 10B).

### Purified PelB-Vpu can selectively associate with CD4

**Fig 11:**
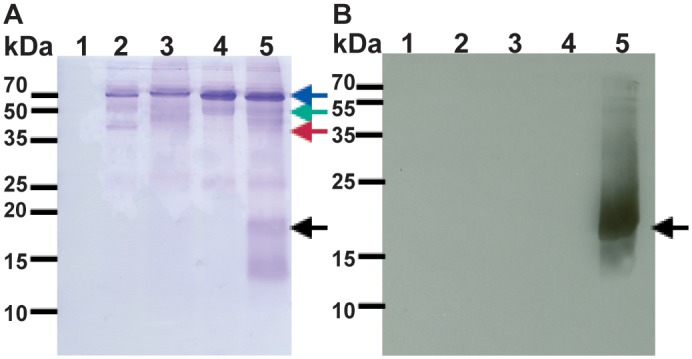
Co-immunoprecipitation of PelB-Vpu with CD4. Purified CD4 TMD + cytoplasmic domain fused to the amino end of MBP (CD4-MBP) was incubated with PelB-Vpu in βDDM micelles. The complex was captured by anti- MBP antibody-conjugated beads and pelleted by centrifugation and analyzed by SDS-PAGE followed by Coomassie staining (**A**) and immunoblot analysis with Vpu antibodies (**B**). Panel **A** depicts all proteins pulled down by only agarose beads (lane 1) and agarose beads conjugated with anti-MBP (lanes 2–5). Anti-MBP, CD4-MBP, MBP and Vpu are denoted by blue, green, red and black arrows respectively. Beads were incubated with purified proteins: CD4-MBP (lane 1), MBP and Vpu (lane 2), CD4-MBP (lane 3), Vpu lane 4), CD4-MBP and Vpu (lane 5).

The interaction with CD4 and its subsequent degradation is one of the well-characterized functions of Vpu. This interaction has been shown to be dependent on a combination of specific amino acids and structural features in the transmembrane and cytoplasmic domains of both proteins. Scrambling the amino acids of the transmembrane domain of Vpu, while preserving its hydrophobicity, decreased its ability to down-regulate surface expression of CD4 in HeLa CD4^+^ cells [56]. This indicates that interactions between the two proteins were disrupted. It has also been shown that specific amino acids in the cytoplasmic domain of CD4 [57] and Vpu are required for their interaction [58]. Furthermore, it was also demonstrated that the formation of α-helical structures in the cytoplasmic domains of both Vpu and CD4 are critical for CD4-Vpu interactions [59]. The transmembrane and cytoplasmic domains of CD4 were expressed as an N-terminal fusion with maltose binding protein (MBP) in BL21(DE3) *E*. *coli* cells. To determine if PelB-Vpu interacts with CD4, the fusion protein in βDDM micelles was purified by metal affinity chromatography and size exclusion chromatography. PelB-Vpu in βDDM micelles was pulled down only when the protein was incubated with the CD4 TMD/cytoplasmic domain-MBP fusion protein (Fig 11A and 11B, lanes 5). This indicates that PelB tag does not abolish interaction between the two proteins as measured by our pull-down assay.

## Conclusions

The requirement to produce substantial quantities of pure, folded, functional and monodispersed protein is a a major factor that hampers elucidation of membrane protein structural models. We demonstrate successful expression of good quantities of HIV-1 Vpu when expressed with the PelB signal sequence appended to the amino end of the protein. The signal peptide was not cleaved from the majority of the protein. The fusion protein was folded, monodispersed and a pull-down assay indicates that the protein retains its ability to interact with the TMD and cytoplasmic tail of CD4 which has been shown to be functionally important for interaction and retention of the protein in cells. We hypothesize that efficient cleavage of the signal peptide was inhibited by the inability of the periplasmic peptidases to access the cleavage site. Nevertheless, this expression strategy can serve as the basis for further Vpu structural studies and possibly a template that will be applicable for other membrane proteins as well.

## Materials and methods

### Target selection, gene optimization and cloning

The sequence of HIV- 1 group M subtype B strain HXB2 Vpu was used. Unfavorable codons for each 82 amino acids in length were replaced using the *E*. *coli* codon usage table [60]. Codon usage bias analysis was based on a set of genes encoding highly expressed proteins of *E*. *coli* according to Sharp and Li [61] and based on the *E*. *coli* codon usage table (ibid). The equivalent set of human cDNAs is described in Table 1. Using Sharp and Li’s methodology [61], we calculated the values of the following parameters: relative synonymous codon usage (RSCU, defined as observed frequency/expected unbiased frequency), the relative codon adaptiveness (*w*, RSCU normalized to the most abundant synonymous codon for each amino acid) and the codon adaptation index (CAI, geometric mean of *w* values over the entire length of the gene). Codons with *w*<0.5 are considered “unfavorable” (i.e. infrequently used).

**Table 1:** Highly-expressed human gene set used in codon usage optimization.

Gene	Description	Accession Number
dclre1A	DNA cross-link repair 1A	NM_014881
dnaJ	Heat shock protein, Hsp40	NM_173650
eef1D	*Homo sapiens* eukaryotic translation elongation factor 1 delta	NM_001130055
eef1G	*H*. *sapiens* eukaryotic translation elongation factor 1 gamma	NM_001404
eif2B3	*H*. *sapiens* eukaryotic translation initiation factor 2B subunit gamma	NM_001166588
eif4E2	*H*. *sapiens* eukaryotic translation initiation factor 4E	NM_004846
eif4ebp1	*H*. *sapiens* eukaryotic translation initiation factor 4E binding protein 1	NM_004095
old35	*H*. *sapiens* 3'-5' RNA exonuclease	AY027528
psmc1	*H*. *sapiens* proteasome 26S subunit, ATPase 1	NM_002802
psmd7	*H*. *sapiens* proteasome 26S subunit non-ATPase, 7	NM_002811
rpl5	*H*. *sapiens* ribosomal protein L5	NM_000969
rpl10	*H*. *sapiens* ribosomal protein L10	NG_012890
rpl11	*H*. *sapiens* ribosomal protein L11	NM_000975
rpl12	*H*. *sapiens* ribosomal protein L12	NM_000976
rpl18	*H*. *sapiens* ribosomal protein L18	NM_000979
rpl19	*H*. *sapiens* ribosomal protein L19	NM_000981
rpl22	*H*. *sapiens* ribosomal protein L22	NM_000983
rpl23	*H*. *sapiens* ribosomal protein L23	NM_000978
rpl26	*H*. *sapiens* ribosomal protein L26	NM_000987
rpl27A	*H*. *sapiens* ribosomal protein L27A	NM_000990
rpl30	*H*. *sapiens* ribosomal protein L30	NM_000989
rpl32	*H*. *sapiens* ribosomal protein L32	NM_000994
rpl34	*H*. *sapiens* ribosomal protein L34	NM_033625
rpl35A	*H*. *sapiens* ribosomal protein L35A	NG_011743
rpl36A	*H*. *sapiens* ribosomal protein L36A	NG_012523
rpl39	*H*. *sapiens* ribosomal protein L39	NG_016250
rplP0	*H*. *sapiens* ribosomal protein lateral stalk subunit P0	NM_001002
rplP1	*H*. *sapiens* ribosomal protein lateral stalk subunit P1	NM_001003
rps2	*H*. *sapiens* ribosomal protein S2	NM_002952
rps4X	*H*. *sapiens* ribosomal protein S4, X-linked	NG_012524
rps6kA4	*H*. *sapiens* ribosomal protein S6 kinase A4	NM_003942
rps6kC1	*H*. *sapiens* ribosomal protein S6 kinase C1	NM_001136138
rps11	*H*. *sapiens* ribosomal protein S11	NM_001015
rps12	*H*. *sapiens* ribosomal protein S12	NM_001016
rps15	*H*. *sapiens* ribosomal protein S15	NM_001018
rps17	*H*. *sapiens* ribosomal protein S17	NM_001021
rps19	*H*. *sapiens* ribosomal protein S19	NG_007080
rps20	*H*. *sapiens* ribosomal protein S20	NM_001023
rps23	*H*. *sapiens* ribosomal protein S23	NM_001025
rps24	*H*. *sapiens* ribosomal protein S24	NM_001142284
rpsA	*H*. *sapiens* ribosomal protein SA	NM_002295

The *vpu* gene optimized for expression in *E*. *coli* was synthesized by overlap extension PCR. Overlapping oligonucleotides, oTMs 638–645 (Table 2) of sizes ranging from 30–40 bases were ordered from Integrated DNA Technologies. The first round of PCR (Roche High Fidelity PCR Kit; cat# 11732641001) to assemble to the gene was done for 25 cycles (initial denaturation: 94°C for 2 min; denaturation: 94°C for 15 s; annealing: 55°C for 30 s; elongation 72°C for 30 s; final elongation: 72°C for 7 min) and was followed by a second round of PCR with external primers oTMs 646 and 647 for 40 cycles for amplification (initial denaturation: 94°C for 2 min; denaturation: 94°C for 15 s; annealing: 58°C for 30 s; elongation 72°C for 30 s; final elongation: 72°C for 7 min). The PCR product was first cloned into a TOPO-TA intermediate cloning vector (Invitrogen; cat# 450641). The *vpu* gene was sub-cloned into a TOPO-TA vector with flanking NcoI and BlpI sites and a C-terminus hexa-histidine tag using oTMs 787 and 788 and was excised from this intermediate vector by digestion with NcoI (NEB; cat# R3193S) and BlpI restriction enzymes (NEB; cat# R0585S). The same pair of restriction enzymes were also used to linearize the pET26(b) vector by Novagen. The restriction digestion reactions were analyzed by agarose gel electrophoresis and the desired DNA bands were excised and purified using a QIAquick gel extraction kit (Qiagen; cat# 28704). The gene was ligated to the pET26(b) vector by performing a T4 DNA ligase reaction (Promega; cat# M1801) and transformed into DH5α electro-competent cells. The resulting bacterial colonies were screened using colony screen PCR using gene specific primers oTMs 662 and 663 and plasmid specific primers, oTMs 856 and 857. DNA sequencing using oTMs 856 and 857 was used to verify the final expression clone, pTM 875.

**Table 2:** Oligonucleotide used in this work.

Primer	Sequence (5’ to 3’)
oTM 638	ggtaccatgcagccgatcccgatcgttgctatcgtagcactggttgtagctatcatc
oTM 639	atgataacgatggaccatacaacgattgcgatgatgatagctacaaccagtgctacgatagcaac
oTM 640	atcgcaatcgttgtatggtccatcgttatcatcgaataccgtaaaatcctgcgtcagcgtaaa
oTM 641	cgatcagacgatcgatcagacggtcgattttacgctgacgcaggattttacggtattcg
oTM 642	atcgaccgtctgatcgatcgtctgatcgaacgtgcagaagactctggcaacgaatc
oTM 643	catttcaaccagtgcagagatttcaccttcggattcgttgccagagtcttctgcacgtt
oTM 644	cgaaggtgaaatctctgcactggttgaaatgggtgttgaaatgggtcaccacgctcc
oTM 645	gtcgaccagatcgtcaacgtcccacggagcgtggtgacccatttcaacacc
oTM 646	ggtaccatgcagccgatcccg
oTM 647	gtcgaccagatcgtcaacgtccc
oTM 662	atgcagccgatcccgatcg
oTM 663	cagatcgtcaacgtcccacgg
oTM 787	ccatgggcatgcagccgatcccgatcgttgc
oTM 788	gctcagcttaatggtgatggtgatggtg cagatcgtcaacgtcccacggagcg
oTM 856	atgaaatacctgctgccgaccgctgc
oTM 857	cccattcgccaatccggatatagttcctcc

### Expression

The expression of PelB-Vpu with the N-terminal PelB signal peptide, along with the C-terminal hexa-histidine affinity tag was driven by a T7 promoter. *E*. *coli* BL21 DE cells were transformed with the expression vector, pTM 875 by electroporation. For protein expression, an overnight culture of BL21 DE cells containing the expression vector was grown in LB media supplemented with 100 μg/mL kanamycin (Phytotechnology lab; cat# K378) and was used to inoculate a fresh LB media with 50 μg/mL kanamycin in a 1:100 dilution. The cultures for expression were shaken at 200 rpm at 37°C to mid-logarithmic phase (OD_600_ ≈ 0.4). The cultures were then moved to 15°C and incubated for additional 30 min with 200 rpm shaking. Finally the cultures were induced for expression with 0.1 mM Isopropyl-β-D-thiogalactoside (IPTG, Roche; cat# 11411446001). For standardization of expression conditions, 5 mL of culture were collected at various time points after IPTG induction and pelleted by centrifugation at 5000 ×*g*, 4°C for 15 min. For detergent extraction screens and subsequent purifications, cells were harvested at 6 h post induction by centrifugation at 5000 ×*g*, 4°C for 15 min. Cell pellets were stored at -80°C until further use.

### Separation of water-soluble and insoluble fractions

Cell pellets from 4 L worth of culture (approximately 12 g) were re-suspended in 100 mL of ice-cold PBS-PI buffer (phosphate buffer saline, PBS, supplemented with EDTA-Free SIGMAFAST^™^ Protease Inhibitor Cocktail tablets, Sigma; cat# S8830). The cells were lysed by double passage through a microfluidizer (Microfluidics Microfluidizer). The lysate was collected and centrifuged at 36,000 ×*g* for 30 min at 4°C. The insoluble fraction, containing the inner and outer membranes among other insoluble materials like protein inclusion bodies, was washed once by repeated re-suspension (50 mL of ice-cold PBS with protease inhibitor cocktail) and centrifugation. The pellet was frozen at -80°C until further use.

### Detergent extraction

Aqueous and non-aqueous fractions from 500 mL cultures were prepared as described above. induced for expression and cells were harvested as pellets as mentioned previously. The pellet containing the non-aqueous proteins was re-suspended in 25 mL of PBS-PI buffer. Samples (4 mL) were then subjected to detergent extraction by addition of the following detergents to final concentration of 1% (w/v): n-dodecyl-β-D-maltoside (βDDM, Glycon Biotech; cat# D97002C), n-decyl-β-D-maltoside (βDM, Anatrace; cat# D322), 3-[(3-Cholamidopropyl) dimethylammonio]-1-propanesulfonate (CHAPS, Mclab; cat# DCPS101), n-octyl β-D-glucopyranoside (OG, Glycon Biotech; cat# D97001C), n-dodecylphosphocholine (DPC, Anatrace; cat# F308S) and n-dodecyl-N,N-dimethylamine-N-oxide (LDAO, Anatrace; cat# D360). The samples were left overnight at 4°C with agitation at 200 rpm and centrifuged at 36,000 ×*g* for 30 min at 4°C on the following day and the supernatant was collected as the detergent soluble fraction. The fractions were analyzed by SDS-PAGE followed by Coomassie staining and western blot.

For large-scale extractions from 4 L cultures, the pellet, containing the membrane fraction, was fully re-suspended in 200 mL PBS supplemented with EDTA-Free SIGMAFAST^™^ Protease Inhibitor Cocktail tablets. βDDM was used for solubilization at a final concentration of 1% (w/v). The protein was extracted and at 4°C overnight with agitation at 200 rpm. The detergent soluble fraction was obtained by collecting the supernatant after centrifugation at 36,000 ×*g* for 30 min at 4°C.

### Purification

A gravity driven column (Bio-Rad Econo-column) containing 60 mL bed volume of TALON metal affinity resin (Clontech laboratories Ltd; Cat# 635503) was equilibrated with binding buffer (20 mM HEPES pH 7.5, 500 mM NaCl, 0.02% βDDM, 5 mM imidazole). The sample was then loaded onto the column, and passed twice. The column was washed with ten bed volumes of wash buffers containing various combinations of salt and imidazole, for example, 20 mM HEPES, pH 7.5 with 500 mM NaCl (Wash I), 500 mM NaCl, 0.02% βDDM (Wash II), 250 mM NaCl, 0.02% βDDM (Wash III) and 250 mM NaCl, 0.02% βDDM, 5% glycerol, 10 mM imidazole (Wash IV) to remove weakly bound proteins. Tightly bound proteins were eluted by application of 3 bed volumes of elution buffer (20mM HEPES pH 7.5, 250 mM NaCl, 0.02% βDDM, 5% glycerol, 300 mM imidazole).

The eluted sample was concentrated by 100 kDa molecular weight cutoff (MWCO) concentrators (Millipore; cat# UFC910024) to approximately 20 times its original volume. Concentrated samples were further purified by size exclusion chromatography (GE Life sciences, Superdex 200 10/300 GL; column volume: 24 mL; fluid phase: 8 mL) using a fast pressure liquid chromatography instrument (FPLC, Pharmacia, Äkta Explorer). The running buffer contained 20 mM HEPES pH 7.5, 250 mM NaCl, 0.02% βDDM, and 5% glycerol. For preparatory separations, a 1 mL sample of concentrated PelB-Vpu was loaded onto the SEC column and chromatography was performed at a flow rate of 0.5 mL/min. The protein elution was detected by absorption at 280 nm. The concentration of protein in the desired peak was determined spectrophotometrically (A_280_) based on the primary sequence of PelB-Vpu; Σ_280_ was calculated with ProtParam web application (http://web.expasy.org/protparam/).

### Determination of PelB-Vpu orientation in the *E*. *coli* inner membranes

A 500 mL LB media, supplemented with 50 μg/mL kanamycin, was inoculated with an overnight culture of BL21 –pTM 875 at a dilution of 1:100. The culture was grown and induced with 0.1 mM IPTG as mentioned above. The culture was allowed to shake at 180 RPM for 3 hr at 15°C post-induction after which the cells were harvested by centrifugation at 5000 ×*g* for 15 min at 4°C. The cells were gently and thoroughly re-suspended in 10 mL of 200 mM TRIS, 500 mM sucrose and 0.1 mM EDTA and were distributed into 1 mL aliquots. Lysozyme was added at a final concentration of 2 mg/mL and mixed 3 times with gentle inversion and incubated on ice for 20 min. Calcium chloride was added at a final concentration of 30 mM and chymotrypsin at 1:1000, 1:100 and 1:50 v/v ratio was added to the appropriate tubes from a stock of 1 mg/mL. Triton X-100 was added at a final concentration of 2% for controls performed with fully lysed cells. The digestion was done at 25°C for 1 hr, with gentle mixing every 15 min. Finally, the cells were lysed by the addition of SDS sample buffer and heating at 100°C before analyzing the results by SDS gel electrophoresis followed by immunoblot and coomassie staining.

### *Co*-immunoprecipitation *with CD4*

A deconstructed version of human CD4 containing the transmembrane and the cytoplasmic domains with an N-terminal fusion of maltose binding protein was expressed in BL21 cells. The protein was extracted and solubilized in buffer containing 0.02% βDDM and purified by metal affinity followed by size exclusion chromatography. MBP was expressed with an N-terminal histidine tag in BL21 strain of *E*. *coli* cells and similarly purified by metal affinity and size exclusion chromatography. 25 μM of the MBP-CD4 fusion was incubated with 50 μM PelB-Vpu for 45 min at 4°C. The reaction was incubated with antibodies against MBP at a dilution of 1:200 for 1 hr followed by 20 μL of Protein A/G PLUS- Agarose immunoprecipitation reagent (Santa Cruz Biotechnology; cat # sc-2003) overnight at 4°C. The agarose beads were pelleted by centrifugation at 500 *×g* for 5 min at 4°C. The beads were washed 4 times with 1 mL of 20 mM HEPES pH 7.5, 250 mM NaCl, 0.02% βDDM and centrifuged for 500 *×g* for 5 min at 4°C. Finally, the beads were resuspended in 1x SDS sample buffer and were analyzed by SDS-PAGE followed by immuno-blot analysis using antibodies against Vpu.

### Gel electrophoresis, staining and western blots

SDS-PAGE using TRIS—based buffers (25 mM TRIS, 200 mM glycine and 3.5 mM SDS) was performed in a Bio-Rad Mini-PROTEAN Tetra Cell. Following electrophoresis, gels were stained by Coomassie brilliant blue, subjected to silver staining, or processed for immuno-blotting. For immuno-blotting, the acrylamide gel was first rinsed with water and equilibrated in a non-denaturing buffer (25 mM TRIS base and 200 mM glycine). The nitrocellulose membrane (Bio-Rad; cat# 162–0112) was also equilibrated in the non-denaturing buffer. The gel and the membrane were sandwiched between extra-thick blot filter papers (VWR; cat# 28298–014) soaked in the non-denaturing buffer and proteins were electro- blotted for 15 min at 15 V using a Bio-Rad Transfer-blot SD Semi-dry Transfer Cell. Following an hr long blocking in PBSTM (PBS, 0.05% Tween 20 and 5% dry milk), the nitrocellulose membrane was further incubated in the presence of the appropriate primary antibody. The membrane was then washed 3×15 min in PBST (PBS and 0.05% Tween 20) prior to a 1 hr incubation with the appropriate secondary antibody conjugated to horseradish peroxidase. Following additional 3x 15 min washes, the nitrocellulose membrane was then soaked in Immunocruz western blotting luminol reagent (Santa Cruz Biotechnology; cat# sc-2048), exposed to a chemiluminescence sensitive film (GE Heathcare; cat# 28906838) for the optimum time of exposure and the film was developed with a Konica SRX-101A system. Anti- Vpu polyclonal antibody, raised in rabbits, was kindly provided by the NIH’s AIDS Reagent Program and was used at a dilution of 1:30,000. Antibodies against AcrA and GroEL were raised in rabbits. Goat anti-rabbit IgG—HRP (Santa Cruz Biotechnology; cat# sc-2923) and rabbit anti-mouse IgG-HRP (Sigma A-9044) were used at a dilution of 1:10,000 in PBSTM.

### Mass spectrometry

We used matrix-assisted laser desorption/ionization-time of flight (MALDI-TOF) Mass spectrometry (MS) to accurately measure the molecular weight of the purified PelB-Vpu protein. Purified PelB-Vpu (1 μL of 10 mg/mL) was added to 4 μl of sinapinic acid matrix solution (67:33 water:acetonitrile (v/v) containing 0.4% trifluoroacetic acid in the total volume, saturated with sinapinic acid). The protein/ matrix mixture (1 μL) was added onto a steel target plate and allowed to dry in air. An external calibration spot prepared in a similar manner was placed near the PelB-Vpu protein spot. The plate was then placed into a Bruker microflex MALDI-TOF mass spectrometer, and spectra were collected in a positive linear mode over a mass range from 1 to 30 kDa. The final results represented the average of 10 separate spectra.

### Circular dichroism

A JASCO J-710 CD spectro-polarimeter was used for measuring the Circular dichroism (CD) spectra of the purified sample at various salt and temperature conditions. The SEC-purified PelB-Vpu (eluted in 20 mM HEPES pH 7.5, 250 mM NaCl, 0.02% βDDM, and 5% glycerol) was concentrated to 10 mg/mL by a 100-kDa concentrator and CD measurement was done on samples containing a final protein concentration of 0.5 mg/mL. A baseline CD spectrum was obtained for the buffer and these values were subtracted from the CD measurement of PelB-Vpu. CD spectra for the protein were recorded from 190 to 260 nm using a 0.1 cm quartz cuvette. To obtain readings for the protein at different salt concentrations, a temperature of 20°C was maintained throughout. Parameters were set at 1 nm data pitch, continuous scanning mode, a scanning speed of 50 nm/min, a response of 4 s, and a spectral bandwidth of 1 nm. Output spectra were generated based on an accumulation of five scans. The molar ellipticity (θ) in deg.·cm^2^/dmol was calculated as described by [62].

### Dynamic light scattering

Dynamic light-scattering (DLS) measurements were performed using a NaBiTec GmbH setup comprising a SpectroSize 302 (Molecular Dimensions) in combination with an S6D microscope (Leica). The purified protein sample was concentrated to 10 mg/mL as described earlier was illuminated in a 2 μl hanging drop using a 24-well crystallization plate (VDX Greased Plate, Hampton Research) covered with siliconized-glass circular cover slides (22 mm; Hampton Research). The well itself was filled with 400 μl SEC running buffer. Prior to the measurement, the protein solution was centrifuged at 18000 *×g*, 30 min, 4°C to remove possible dust and other suspended particles. All measurements were done at 20°C. Ten consecutive measurements, each with an integration time of 20 s, were averaged. Hydrodynamic size of the particles was estimated with the instrument software using the following parameters: refractive index 1.33, viscosity 1.006, shape factor 1.0 and hydrated shell 0.2 nm.
